# Pan-American Medical Congress

**Published:** 1893-10

**Authors:** 


### PAN-AMERICAN MEDICAL CONGRESS.

---

The assembling of the Pan-American Medical Congress in Washington, September 5th, 6th, 7th and 8th, was a notable gathering of eminent physicians from almost every American country.

The support of the Government of the United States, both moral and financial, the hearty co-operation of all the other American governments, and the official recognition given by President Cleveland in opening the Congress are all circumstances which indicate the wonderful growth which medicine has made in the minds of the American people, and foreshadow still greater results in the future. May we not hope that it presages the creation of a Department of Public Health, in the early future, to be presided over by a cabinet officer of equal rank with other cabinet officials?

For the work thus far accomplished great credit is due Dr. Charles A. L. Reed of Cincinnati. It was he who introduced a resolution at the meeting of the American Medical Association in Washington in May, 1891, which initiated the movement that ripened into the recent most successful meeting. An earnest effort was made to bring about scientific co-operation in quarantine and sanitary regulations and greater uniformity in the standards of medical education throughout the Western Hemisphere.

Much real scientific work was done and many valuable contributions were made to medical literature.

The City of Mexico was selected as the next place of meeting, but the date was left to the International Executive Committee with the understanding, however, that the next meeting should be held either in 1896 or 1897.

